# Blood–brain-barriers in aging and in Alzheimer’s disease

**DOI:** 10.1186/1750-1326-8-38

**Published:** 2013-10-22

**Authors:** Fernanda Marques, João Carlos Sousa, Nuno Sousa, Joana Almeida Palha

**Affiliations:** 1 Life and Health Sciences Research Institute (ICVS), School of Health Sciences, University of Minho, Campus Gualtar, Braga 4710-057, Portugal; 2 ICVS/3B’s - PT Government Associate Laboratory, Braga/Guimaraes, Portugal

**Keywords:** Aging, Alzheimer’s disease, Blood–brain barrier, Blood-cerebrospinal fluid barrier, Cerebrospinal fluid, Choroid plexus

## Abstract

The aging process correlates with a progressive failure in the normal cellular and organ functioning; these alterations are aggravated in Alzheimer’s disease (AD). In both aging and AD there is a general decrease in the capacity of the body to eliminate toxic compounds and, simultaneously, to supply the brain with relevant growth and nutritional factors. The barriers of the brain are targets of this age related dysfunction; both the endothelial cells of the blood–brain barrier and the choroid plexus epithelial cells of the blood-cerebrospinal fluid barrier decrease their secretory capacity towards the brain and their ability to remove toxic compounds from the brain. Additionally, during normal aging and in AD, the permeability of the brain barriers increase. As such, a greater contact of the brain parenchyma with the blood content alters the highly controlled neural environment, which impacts on neural function. Of interest, the brain barriers are more than mere obstacles to the passage of molecules and cells, and therefore active players in brain homeostasis, which is still to be further recognized and investigated in the context of health and disease. Herein, we provide a review on how the brain barriers change during aging and in AD and how these processes impact on brain function.

## Introduction

Increases in lifespan over the last decades have, unfortunately, not been matched by improvements in the mental health span. While some individuals age “healthily”, others present accelerated cognitive decline. Persons over the age of 65 have high risk of developing Alzheimer’s disease (AD), the most common type of dementia. AD affects approximately 28 million people worldwide and it is 1 in 85 persons (or 106 million people) will suffer from AD [1]. The need to counter these disorders, based on improved mechanistic understanding of their etiopathogenesis cannot, therefore, be underestimated.

The main pathological features of AD are the extracellular deposition of amyloid β peptide (Aβ) into plaques and the formation of intracellular tangles composed of hyperphosphorylated Tau protein [2]. Various lifetime parameters, such as chronic stress and exposure to inflammatory stimuli have been suggested to predispose individuals to AD, and several molecular pathways have been implied in the disease [3,4]. In this review we will focus on the involvement of the brain barriers in aging and in AD. This topic is still poorly investigated, especially in normal aging, but is of relevance given the ability of the brain barriers to maintain and regulate the environment for the normal neuronal activity. Thus, alterations in the barriers’ morphology, secretome and functioning can compromise central nervous system (CNS) homeostasis. A key message from this review is that the brain barriers are not mere obstacles to the passage of molecules, cells and drugs into and out of the brain (which by itself is of relevance for clearance of Aβ peptides); in fact, they actively contribute to brain homeostasis and display specific responses to events that occur in the periphery and in the brain parenchyma, which should be taken into consideration in understanding diseases of the CNS.

### The barriers of the brain

CNS homeostasis is essential for the proper functioning of brain cells. The blood–brain barriers participate in CNS homeostasis by preventing the brain from being exposed to the constant oscillations in the concentration of blood constituents and by transporting nutrients and products from brain metabolism in and out of the brain, respectively. Two main barriers separate the CNS from the periphery: the blood–brain barrier (BBB) and the blood-cerebrospinal fluid barrier (BCSFB) [5]. While the BBB has been well recognized for long; the BCSFB is more rarely mentioned, which is to regret when considering its functions, which include producing most of the cerebrospinal fluid (CSF).

The existence of the brain barriers is in part responsible for the initial concept that the brain is an immune-privileged site, with restricted passage of immune cells into the brain. In fact, even though in a healthy person under physiological conditions, immune cell migration across the brain barriers is low, some migration exists and is required for the immune surveillance of the CNS [6,7]. However, during normal aging and in several diseases of the CNS, such as multiple sclerosis and AD, changes in blood composition, brain inflammation and the facilitated entrance of immune cells through the brain barriers can potentially cause neuronal damage and cognitive dysfunction [8-10].

While the brain barriers protect the neural milieu from drastic concentration changes in blood molecules such as nutrients and ions, the brain still senses changes in specific blood constituents to rapidly respond accordingly. Specific brain regions, called circumventricular organs (area postrema, median eminence, pineal gland, posterior pituitary, subfornical organ, subcomissural organ, and the vascular organ of the lamina terminalis) are deprived of BBB and, therefore, continuously monitor the blood composition. In these areas the protection of the brain parenchyma is ensured by tanycytes [11].

### The blood–brain barrier

**Figure 1:**
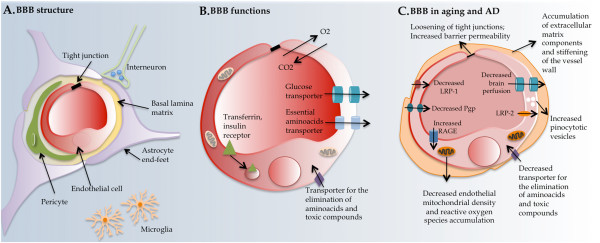
**Neurovascular unit composition, function and alterations in aging and in AD. (A)** The BBB is formed by the tight junctions that connect the endothelial cells of the brain capillaries. These are surrounded by a basal lamina, pericytes and by astrocytes end-feets, and may as well interact directly or indirectly with neurons. **(B)** The endothelial cells of the BBB contain in the luminal and abluminal sides transporters and receptors. **(C)** Several alterations are observed at the endothelial cells of the BBB during aging and in AD.

The BBB is formed by tight junctions present between the endothelial cells of the capillaries that perfuse the brain parenchyma. On a larger organizational level, the basal lamina, astrocytic end-feets and pericytes that surround the central BBB core, altogether, constitute what is designated as the neurovascular unit (Figure 1A). The total length of capillaries in the human brain is of approximately 600 Km, with a capillary surface area of about 20 m^2^[12]. In fact, almost every neuron is perfused by its own capillary, which highlights the dimension of this interface between the blood and the brain parenchyma.

Except for small lipophilic molecules such as O_2_ and CO_2_, which diffuse freely across endothelial cells along their concentration gradient, access through the brain barriers of blood-born molecules requires the presence of specific transporter or receptor systems. Nutrients like glucose and amino acids enter the brain through specific transporters, while molecules such as insulin, leptin and transferrin are transported by receptor-mediated endocytosis [13,14] (Figure 1B). Conversely, similar mechanisms constitute a way out of the brain for molecules produced by brain cells metabolism; both through passive diffusion and via transporters and receptors located on the “brain side” of the endothelial cell, such as for glutamine and Aβ peptides [15].

### Blood-cerebrospinal fluid barrier

**Figure 2:**
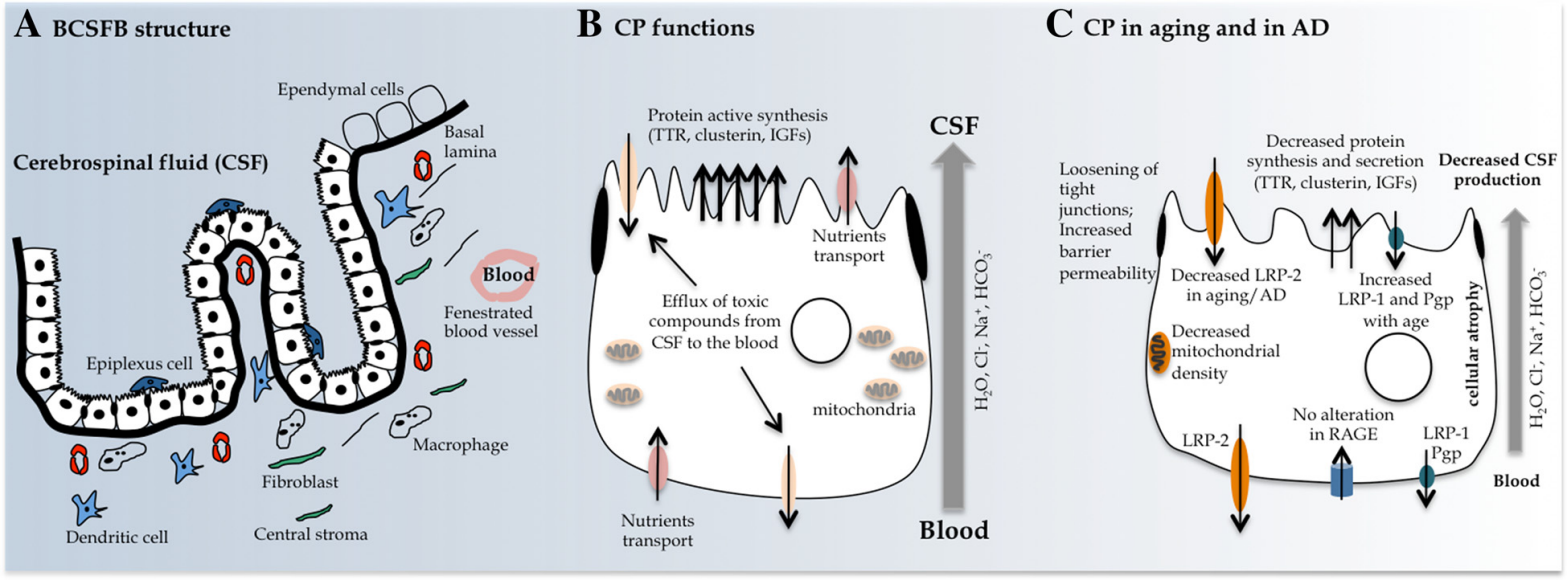
**BCSFB composition, function and alterations in aging and AD. (A)** The CP is formed by a monolayer of epithelial cells originating in the ependymal cells that line the brain ventricles, forming a closed structure – the stroma. **(B)** CP produces CSF. CP epithelial cells contain transporters and receptors in both the basolateral and apical sides. **(C)** Several alterations are observed at the epithelial cells of the BCSFBB during aging and in AD.

The BCSFB is formed by the choroid plexus (CP) epithelial cells. The CP is phylogenetically and ontogenetically conserved. The CP develops early during embryogenesis and already constitutes a functional barrier within the first weeks of gestation [16,17]. Before this barrier is formed, the neuroependymal cells lining the ventricular wall are connected by strap junctions, halting the passage of large molecules into the brain parenchyma [17]. The CP is positioned within the ventricles of the brain: one in each lateral, one in the third and one in the fourth. Grossly, the CP is a lobulated structure formed by a unique and continuous line of epithelial cells originating from the ependymal wall of the ventricles, which floats in the CSF space. These epithelial cells are bound to each other by tight junctions and rest on a basal lamina and on a central core formed by connective and highly vascularized tissue (Figure 2A). The apical side of the epithelial cells faces the CSF and contains numerous villosities, while the basolateral side faces the blood, lying in the stroma in contact with several capillaries. Of notice, the capillaries that irrigate the CP are fenestrated, i.e. at the CP there is no BBB. In addition to the fenestrated capillaries, dendritic cells, fibroblasts and macrophages populate the central core of the CP stroma. Although the passage of molecules and cells is possible in the vascularized stroma, these do not reach the CSF through paracellular transport due to the tight junctions between CP epithelial cells. Ultrastructurally, the epithelial cell contains numerous mitochondria, Golgi apparatus, smooth endoplasmic reticulum and lysosome-like vesicles, which demonstrates that it is a structure with great synthetic capacity. The main CP function is the production of CSF [18] (Figure 2B). The CSF is a clear, slightly viscous liquid with few cells and a protein concentration about 10 times lower than that of the blood [19]. An adult human contains approximately 150 ml of CSF filling the ventricles, the subarachnoid space and the spinal cord, and these are renewed 3–4 times daily, testifying to the great secretory capacity of the CP epithelium. Drainage of CSF occurs at the arachnoid villus in the venous sinus.

By influencing the CSF composition, which ultimately impacts on the brain parenchyma, the CP may interfere with brain homeostasis in health and in disease.

### The brain barriers in aging and in Alzheimer’s disease

The brain has two fluid environments: the brain interstitial fluid, which surrounds the neurons and glia, and the CSF. Interaction between the periphery and the brain parenchyma has, therefore, to occur through the CSF and/or through the neurovascular unit. Both the BBB and the BCSFB contain transporters and receptors in the basolateral and apical sides, and therefore are able to transport molecules into and out of the brain, and to convey receptor-mediated signals, similarly in both directions. What is striking and still far from being fully understood is how the brain barriers themselves respond to stimuli, as recently shown by alterations in the CP transcriptome in response to peripheral inflammatory stimuli [20,21], and in the BBB transcriptome similarly in response to systemic inflammation and to infection [22]. This brings the brain barriers to a novel level of complexity since changes in their functions may be critical to control/prevent/treat diseases of the CNS.

During aging and in AD several alterations are observed in the cellular elements of the neurovascular unit and in the CP epithelia. At the neurovascular unit these consist of focal necrosis of the cerebral endothelium, accumulation of extracellular matrix components in the vascular basement membrane, decreased endothelial mitochondrial density, increased pinocytotic vesicles, loosening of tight junctions, changes in the astrocytic endfeet and stiffening of the vessel wall (with concomitant loss of elasticity that affects brain perfusion) [23,24] (Figure 1C). Furthermore, normal aging and AD are accompanied by a significant decrease in microvessel density [25]. Sensitive neuroimaging methods confirm aging-related regression in global and regional measures of cerebral blood flow (∼4 mL/min/year), cerebral metabolic rate for oxygen, glucose oxidation and cerebral blood volume [26]. Interestingly, deficiency of perycites (one of the elements of the neurovascular unit) has been shown to compromise the integrity of the BBB and lead to brain hypoperfusion resulting in secondary neurodegenerative changes [27,28]. Similarly, the aged human CP exhibits cellular atrophy, decreased CSF production, enzymatic and metabolic activities, and impaired capacity for the efflux and clearance of molecules [29-31] (Figure 2C).

The aged CP epithelial cell cytoplasm becomes rich with Biondi ring tangles and lipofuscin deposits, which is also observed in the CP of AD patients [32]. Additionally, irregular and flattened basement membrane thickening is also observed in the aged CP [29]. The stroma also thickens and contains collagen fibers, hyaline bodies and calcifications while the infiltrating arteries become thicker and fragmented [29,30].

With respect to CSF dynamics, studies in humans and in rodents [33,34] revealed decreased CSF production and turnover in the aged brain. Interestingly, Aβ1-40 and Aβ1-42 concentrations in the cortex and hippocampus were also reported to increase from 3 to 30 months [33], suggesting that the decrease in the CSF turnover and the continuous Aβ brain accumulation are contributing to AD pathology [33,35]. A general feature of aging that is accelerated in AD is an increase in the barriers permeability, as revealed by higher protein leakage from the blood into the CSF [31,36] (Figures 1 and 2C).

Recognition that the barriers’ properties and metabolism change with age and are subject of alterations in response to specific stimuli, make them physiologically interesting in the context of brain function in “healthy“ aging and in disease. We will next briefly specify two examples in which changes at the brain barriers are of relevance for aging and AD: i) the transport of the Aβ peptide since accumulation of Aβ may result from increased Aβ production or decreased Aβ efflux out of the brain [30,37], and ii) the response of the barriers to inflammation, not only given the inflammatory component that is present in the AD brain, but also because peripheral inflammatory stimuli have been shown to alter the barriers homeostasis with respect to processes such as iron regulation, which impacts in AD [38].

### Aβ transport through the brain barriers in Alzheimer’s disease

One of the pathological hallmarks of AD is the increased production and accumulation of Aβ peptides in the brain. These peptides result from the sequential cleavage of the transmembrane amyloid precursor protein (APP). Due to an unidentified combination of events, Aβ monomers can aggregate into oligomers, and then deposit in the form of extracellular amyloid plaques in different regions of the brain [39,40]. The accumulation of these peptides can result both from the increased production but also from a decrease in their excretion through the brain barriers [41]. Thus, excretion of Aβ out of the brain represents a relevant mechanism of the disease and a therapeutic target. Various transporter systems seem to operate at the brain barriers. Although not specific for Aβ, these are involved in receptor-mediated flux of Aβ: the low-density lipoprotein receptor-related protein (LRP), the receptor for advanced glycation end products (RAGE), the receptor glycoprotein330 (gp330)/megalin (LRP-2)-mediated transcytosis, and the ATP-binding cassette, sub-family B (MDR/TAP), member 1 (ABCB1 also known as P-glycoprotein or Pgp) [15,23,42]. While LRPs and ABCB1 mediate the efflux of Aβ from the brain to the periphery, RAGE has been implicated in the Aβ influx to the brain from the periphery [15,37]. The expression of both types of receptors at the BBB is altered with age; the expression of the Aβ efflux transporters is decreased while that of the Aβ influx transporter is increased, adding to the amyloid burden in the brain [15,43]. LRP-1 and ABCB1 staining of microvessels revealed that there is an age-dependent loss of capillary LRP-1 and ABCB1 and that the expression of LRP-1 correlated negatively with the expression of RAGE [43], which seems to progressively contribute to Aβ accumulation in aging (Figure 1C). In addition, single-nucleotide polymorphisms in the ABCB1 gene found in AD patients may be related to changes in ABCB1 function at the BBB. As such, genetic variations in ABCB1 might contribute to the progression of Aβ deposition in the brain [44,45].

As for the BCSFB, LRP-2 has been found to decrease with age, which supports decreased clearance of Aβ [46]. Of interest, a recent study in old rats confirmed reduced LRP-2 but showed an increase in the transcription of the Aβ efflux transporters LRP-1 and ABCB1 and no changes in RAGE expression [47] (Figure 2C). These observations in efflux transport regulation are on the opposite direction from those reported in the BBB. Therefore, it is the overall balance of these transporters at both barriers that finally determines the net flow of Aβ in aging and in AD.

Still related with Aβ removal from the brain, three CSF circulating proteins deserve specific consideration: transthyretin (TTR), clusterin (or apolipoprotein J), and insulin-like growth factor 1 (IGF1).

TTR [48] is a 55-kDa tetrameric protein synthesized mainly by the liver and by the CP [49,50] from where it is secreted into the blood and the CSF, respectively. TTR synthesis represents 20% of the total protein synthesized by the CP. TTR is a plasma and CSF carrier for thyroxine and retinol (vitamin A) [when bound to the retinol-binding protein (RBP)] [51]. Although TTR has been initially proposed to be essential to mediate thyroid hormone and retinol transfer into the tissues, particularly into the brain and across the BCSFB, studies with a TTR-null mouse strain showed that TTR is neither necessary for thyroid hormones entry into and distribution within the brain and other tissues, nor for the maintenance of a euthyroid status [51,52] or for retinol delivery to the tissues [53]. However, both TTR ligands may, themselves, be of relevance in aging and in AD, as exemplified by reports on the ability of retinoids to diminish amyloid aggregation and to improve cognition [54,55]. As for Aβ, TTR is able to bind it both *in vitro* and *in vivo*[56] and, by sequestering Aβ in the CSF, TTR has been suggested to prevent amyloid plaques formation [57-59] and to mediate CP clearance of Aβ. Studies in which TTR-null mice were crossed with animal models of AD have led to contradictory results on whether, *in vivo*, the absence of TTR ameliorates [60,61] or accelerates [62] the AD-like phenotype. Interestingly, the absence of TTR, *per se*, has been shown to accelerate the cognitive decline associated with aging [63]. Studies in humans found decreased TTR levels in the CSF of AD patients [64], which may result from the decreased CP secretory activity described to occur with aging [26]. Furthermore, TTR is also decreased in the blood of individuals with mild cognitive impairment and with AD [65]. These observations suggested that mutated forms of TTR, with decreased affinity to Aβ, could be associated with AD; however, no mutations in TTR were found associated with the disease [66].

Clusterin is another blood and CSF carrier of soluble Aβ [67] synthesized by the CP [68]. Genome-wide association studies found clusterin linked to AD [69] and some studies suggested that its circulating increased levels could be part of a panel of markers of the disease [70]; the latter is however controversial as other studies showed no association [71]. Clusterin levels in AD brain are higher than those in control subjects: in a recent immunohistochemistry characterization, a unique and specific association between clusterin and Aβ1-40 (but not with Aβ1-42) plaques was observed in the cerebral cortex and in the cerebrovasculature of AD subjects [72]. As such, clusterin-bound Aβ1-40 seems prone to deposit in the AD brain. On the contrary, binding to clusterin may facilitate its transport across the BBB and the BCSFB through LRP-2 [68,73]. Therefore, the factors that determine whether clusterin is protective or detrimental in AD remain to be clarified.

IGF-1 is among the proteins synthesized and secreted by the CP described as neuroprotective in the context of AD, given its ability to participate in the clearance of Aβ out of the brain. In accordance, treatment of mice over-expressing mutant APP/ presenilin-1 (APP/PS1) with IGF-1 reduced brain Aβ burden [74], an effect suggested to be mediated by interaction with the LRP2 Aβ clearance pathway [75], but also related to an increase in the concentration of other Aβ carrier proteins (including TTR and clusterin). As a promiscuous receptor, LRP-2 is also able to transcytose insulin and IGF-1 [76,77]. Therefore, IGF-1 directly produced by the CP or originating from the blood may enhance the transport of Aβ out of the brain [74,77]. Since levels of APP are not modified after IGF-1 therapy, and *in vitro* data indicate that IGF-1 increases the transport of Aβ/carrier protein complexes through the BCSFB, it seems that IGF-1 favors elimination of Aβ from the brain, supporting a therapeutic use of this growth factor in AD [77].

### Blood–brain barriers and inflammation: impact on aging and Alzheimer’s disease

In a healthy person, immune cell migration through the brain barriers is low. However, as it happens in neuroinflammatory diseases, an increased number of immune cells reach the CNS during aging and in AD [78,79]. Both aging and AD are associated with altered immune response, namely with an increase in the production of inflammatory mediators.

Two levels of discussion seem to be relevant with respect to AD: the inflammatory response that is present in the vicinity of the amyloid plaques and the one that occurs at the level of the brain barriers, especially at the BBB, since considerable evidence implicates vascular inflammation in aging and in AD. Although cerebral vascular endothelial cell dysfunction and leukocyte transmigration across the BBB are described as early events in the development of AD, it is difficult to delineate whether they represent a cause or a consequence of the disease; but they certainly contribute for the perpetuation of the diseased brain [9,10].

In what concerns AD and inflammation, it is known that Aβ enhances microglia and macrophage activation and induces secretion of proinflammatory cytokines and chemokines [80,81]. Recent neuropathological studies showed a close relationship between fibrillar Aβ deposits, inflammation and neurodegeneration in relatively early stages of AD [82]. With respect to blood vessels and inflammation in AD, Aβ appears directly involved in the degeneration of both the larger perforating arterial vessels and in the cerebral capillaries that constitute the BBB. The cerebrovascular pathology in AD also encompasses macro- and micro-infarctions, hemorrhages, lacunas, and ischemic white-matter changes [83,84]. Additionally, immunofluorescent analysis of the cerebrovasculature in AD mouse models demonstrates significant increases in thrombin, hypoxia-inducible factor 1α, interleukin-6 (IL-6), monocyte chemoattractant protein-1 (MCP-1), matrix metalloproteinases and reactive oxygen species [85]. Additionally, isolated brain microvessels obtained from AD patients present high levels of both cell-associated and soluble cytokines and chemokines including interleukin-1 beta, IL-6, interleukin-8, tumor necrosis factor -, transforming growth factor-beta and MCP-1, when compared to age-matched non-AD controls [86-88]. Interestingly, the protective effect of serum IGF-1 in the regulation of brain Aβ levels is antagonized by tumor necrosis factor-, a pro-inflammatory cytokine putatively involved in dementia and aging [74].

Peripheral inflammatory conditions may also trigger the barriers and modulate their response in aging and in CNS diseases. Of interest, whether sustained or repeated exposure to inflammation increases susceptibility to diseases of the CNS is still to be determined. Accordingly, an increasing body of evidence supports the relevance of brain barriers-specific responses to inflammation. While single acute exposure to peripheral lipopolysaccharide (LPS) results in a rapid and transient response of the CP transcriptome [20], continuation of the same stimuli in a repeated chronic mode results in a more attenuated CP response [21]. Similarly, the endothelial cells of the BBB have both constitutive and induced expression of receptors for different proinflammatory ligands that have the ability to stimulate various signaling pathways that are equally responding to peripheral LPS in the CP [21,22]. Of notice, when LPS is administered to the Tg2576 APP mouse model of AD, an increased level of brain IL-6 is observed. Moreover, the permeability of the BBB is increased suggesting vulnerability of the BBB to inflammation in this animal model of AD [89]. Interestingly, however, is the finding that intracranial administration of LPS to promote neuroinflammation results in a reduction in Aβ burden due to microglial activation [90].

When analyzing the barriers response to LPS/peripheral inflammation, one of the genes whose expression was found altered in the CP and in the BBB is that encoding for lipocalin 2 (LCN2) [91], an acute phase response protein that binds iron-loaded bacteria siderophores [92,93]. This initial finding led to a detailed analysis of iron homeostasis in the CP, which revealed that the CP is able to regulate iron homeostasis in the brain, a novel concept of regional iron homeostasis [38]. Iron is a mediator of oxidative stress and accumulates in the AD brain. Of notice, the APP gene has an iron-response element in its 5′untranslated region [94] and was shown to have iron-export ferroxidase activity [95]. Also recently, iron was shown to induce Aβ aggregation since the presence of Fe^3+^ during the Aβ aggregation process blocks the fusion of fibrils into the less toxic amyloid deposits and favors the stabilization of more toxic intermediate forms [96]. Iron access to the brain occurs by transferrin-mediated endocytosis both at the BBB and at the BCSFB. It is therefore likely that changes in brain barrier homeostasis, either with respect to transport system or through altered transcriptome may predispose the brain to increased iron-mediated oxidative stress. Of interest, decreased levels of CSF LCN2 were found in individuals with mild cognitive impairment [97,98], and mice lacking LCN2 presented cognitive impairment [99]. Furthermore LCN2 was shown to enhance the toxicity of glutamate and Aβ [98] and to regulate neuronal morphology and excitability in the hippocampus and in the amygdala upon acute stress [100,101], which could have impact on the neuronal dysfunction that is observed during aging and in response to AD.

## Conclusions

Here we highlighted that the communication between the periphery and the brain, through the brain barriers, is compromised in aging and in AD. Not only alterations in the brain barrier transport mechanisms may influence clearance of Aβ out of the brain, but also homeostatic mechanisms present at the brain barriers, such as their secretome and receptor-mediated signaling, can participate in the neuroinflammation observed in AD. While the literature is revealing that the barriers are active participants in brain homeostasis, additional studies are still required to fully understand on how the barriers function is altered in aging and contribute to neurodegenerative diseases such as AD.

## Abbreviations

Aβ: Amyloid beta; ABCB1: ATP-binding cassette sub-family B (MDR/TAP), member 1; AD: Alzheimer’s disease; APP: Amyloid precursor protein; APP-Tg: APP transgenic mice; BBB: Blood brain barrier; BCSFB: Blood cerebrospinal fluid barrier; CNS: Central nervous system; CP: Choroid plexus; CSF: Cerebrospinal fluid; IL-6: interleukin-6; IGF-1: Insulin growth factor 1; LCN2: Lipocalin 2; LPS: Lipopolysaccharide; LRP: Lipoprotein receptor-related protein; MCP-1: Monocyte chemoattractant protein-1; Pgp: P-glycoprotein; RAGE: Receptor for advanced glycation end products; gp330: Receptor glycoprotein330; sAβ1-40: Soluble Aβ1-40; TTR: Transthyretin.

## Competing interests

The authors declare that they have no competing interests.

## Authors’ contributions

JAP coordinated the work and contributed to draft the manuscript together with FM, JCS and NS. All authors read and approved the final manuscript.

## Authors’ information

Fernanda Marques, Nuno Sousa and Joana Almeida Palha: Participate in EURON - European Graduate School of Neuroscience.
